# Author Correction: A transition phase in late mouse oogenesis impacts DNA methylation of the early embryo

**DOI:** 10.1038/s42003-024-06157-x

**Published:** 2024-04-18

**Authors:** Kristeli Eleftheriou, Antonia Peter, Ivanna Fedorenko, Katy Schmidt, Mark Wossidlo, Julia Arand

**Affiliations:** Department of Cell and Developmental Biology, Center of Anatomy and Cell Biology, Medical University of Vienna, 1090 Vienna, Austria

**Keywords:** Oogenesis, DNA methylation

Correction to: *Communications Biology* 10.1038/s42003-022-04008-1, published online 2 October 2022

In this article the grant number 10.55776/P33984 relating to Austrian Science Fund for M.W. was omitted. The original article has been corrected.

