# Supplementary material for: A transition phase in late mouse oogenesis impacts DNA methylation of the early embryo
Source: Commun Biol. 2022 Oct 2;5:1047. doi: 10.1038/s42003-022-04008-1 (PMC9527251; doi:10.1038/s42003-022-04008-1)
Supplement: Supplementary file 2 — Supplementary Information [file 42003_2022_4008_MOESM2_ESM.pdf]

Supplementary Information

A transition phase in late mouse oogenesis impacts DNA methylation of the early embryo

Kristeli Eleftheriou[1], Antonia Peter[1], Ivanna Fedorenko[1], Katy Schmidt[1], Mark Wossidlo[1,*] & Julia Arand[1]

*corresponding author: mark.wossidlo@meduniwien.ac.at

Affiliation

[1]Department of Cell and Developmental Biology, Center of Anatomy and Cell Biology, Medical University of Vienna, 1090, Vienna, Austria

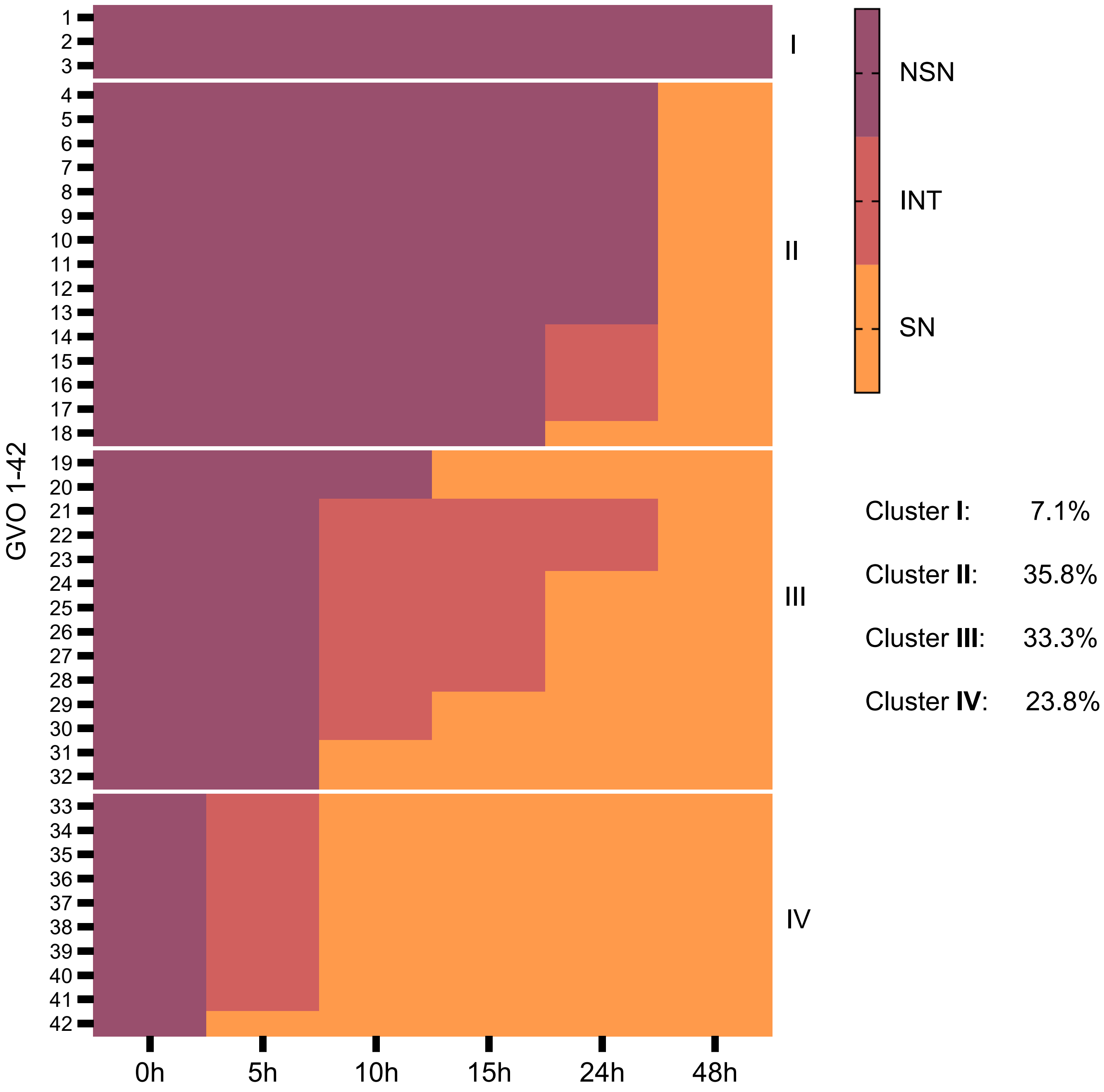

Supplementary Figure 1: *Ex vivo* transition of NSN-GVOs to SN-GVOs

Heatmap representing the chromatin status of 42 NSN-GVOs (two independent experiments) over a timespan of 48 h, while incubating under culture conditions inhibiting *in vitro* maturation (medium supplemented with IBMX). Four different clusters can be discriminated (I-IV). Cluster I, where GVOs stay in the NSN-state; Cluster II, in which NSN-GVOs transition after 15 h into the SN-state; cluster III, in which they transition after 5 h and cluster IV, in which they start transitioning before 5 h. In 59% of cases, an INT-GVO state was observed during the transitioning process.

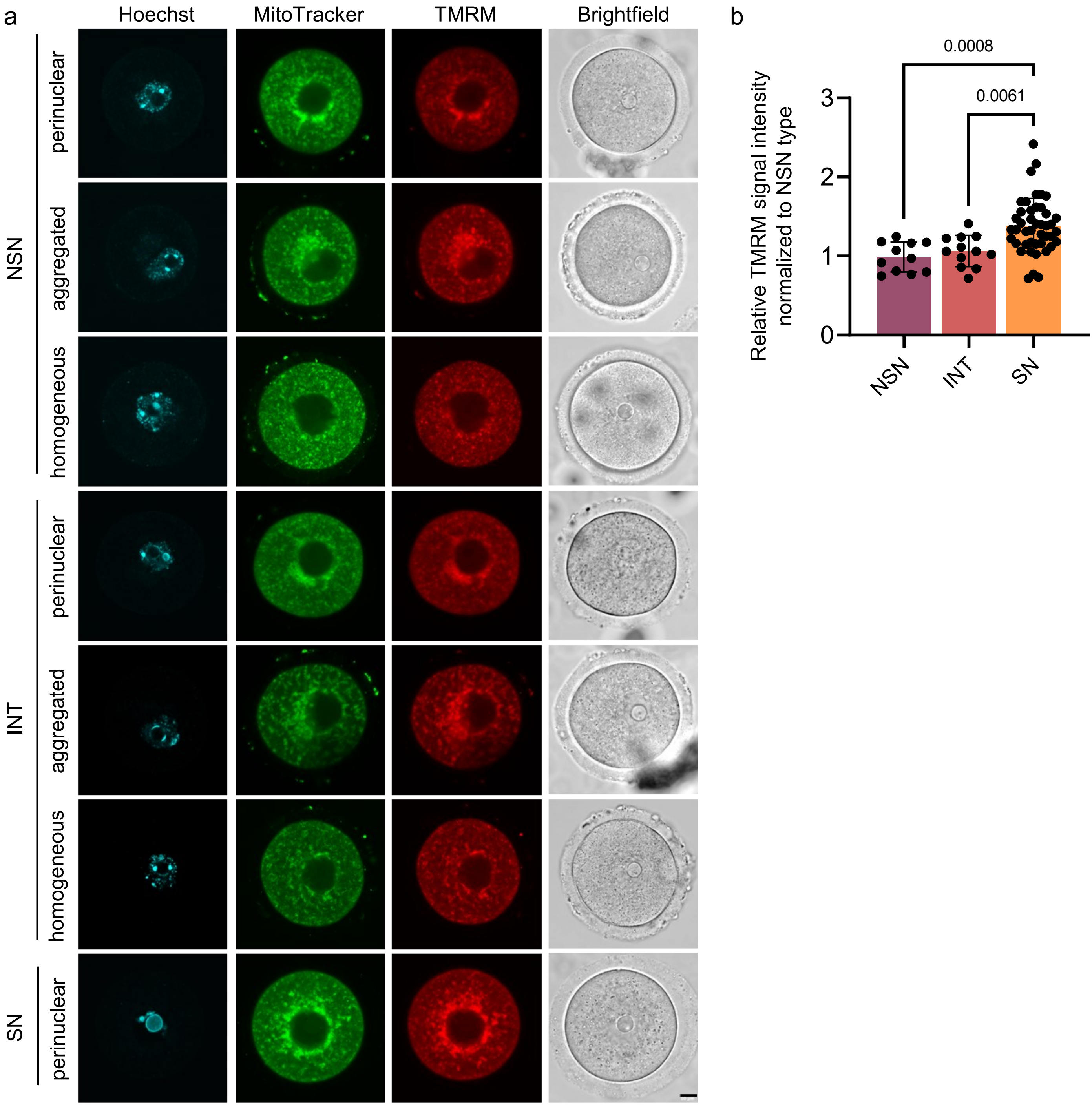

Supplementary Figure 2: Changes of mitochondrial localization and activity during the NSN- to SN-transition

(a) Representative confocal images of the three types of GVOs stained with Hoechst 33342, MitoTracker Green and TMRM. Three mitochondrial distributions can be observed: perinuclear, aggregated and homogeneous. Scale bar = 10µm.

(b) Relative TMRM-signal intensity in NSN-, INT-, and SN-GVOs. Each dot represents a single oocyte (n=11 for NSN, n=12 for INT and n=44 for SN; data are derived from three independent experiments). Statistical significance was calculated using one-way ANOVA with Tukey's multiple comparison test; data are represented as mean ± SD.

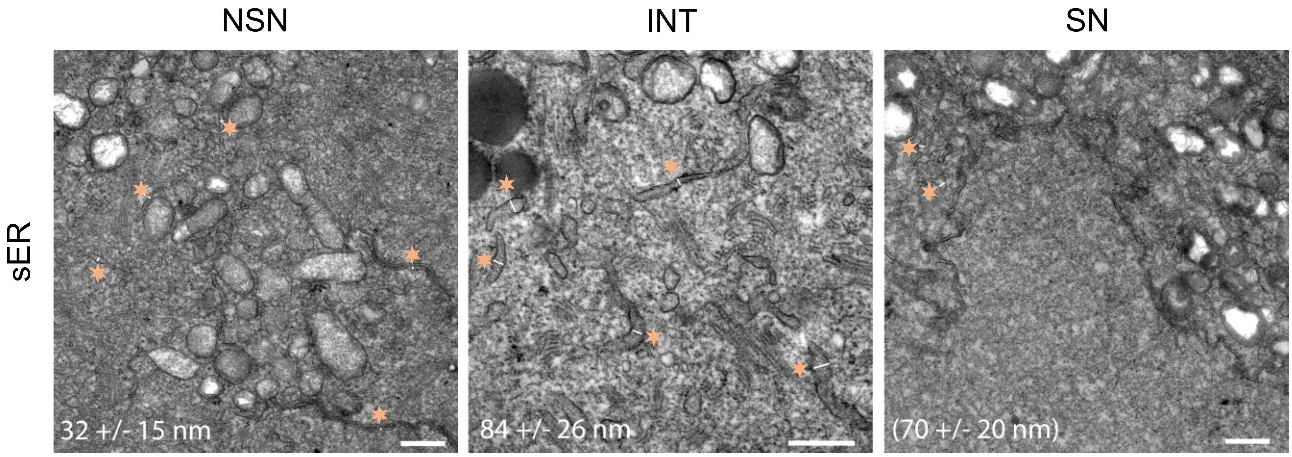

Supplementary Figure 3: Occurrence of the smooth endoplasmic reticulum in the different types of GVOs

Transmission electron microscopy images of NSN-, INT-, and SN-GVOs. Smooth endoplasmic reticulum is marked with a star and the width was measured at the site marked with a white line. Mean width +/- SD is given in the images. Scale bar = 500 nm.

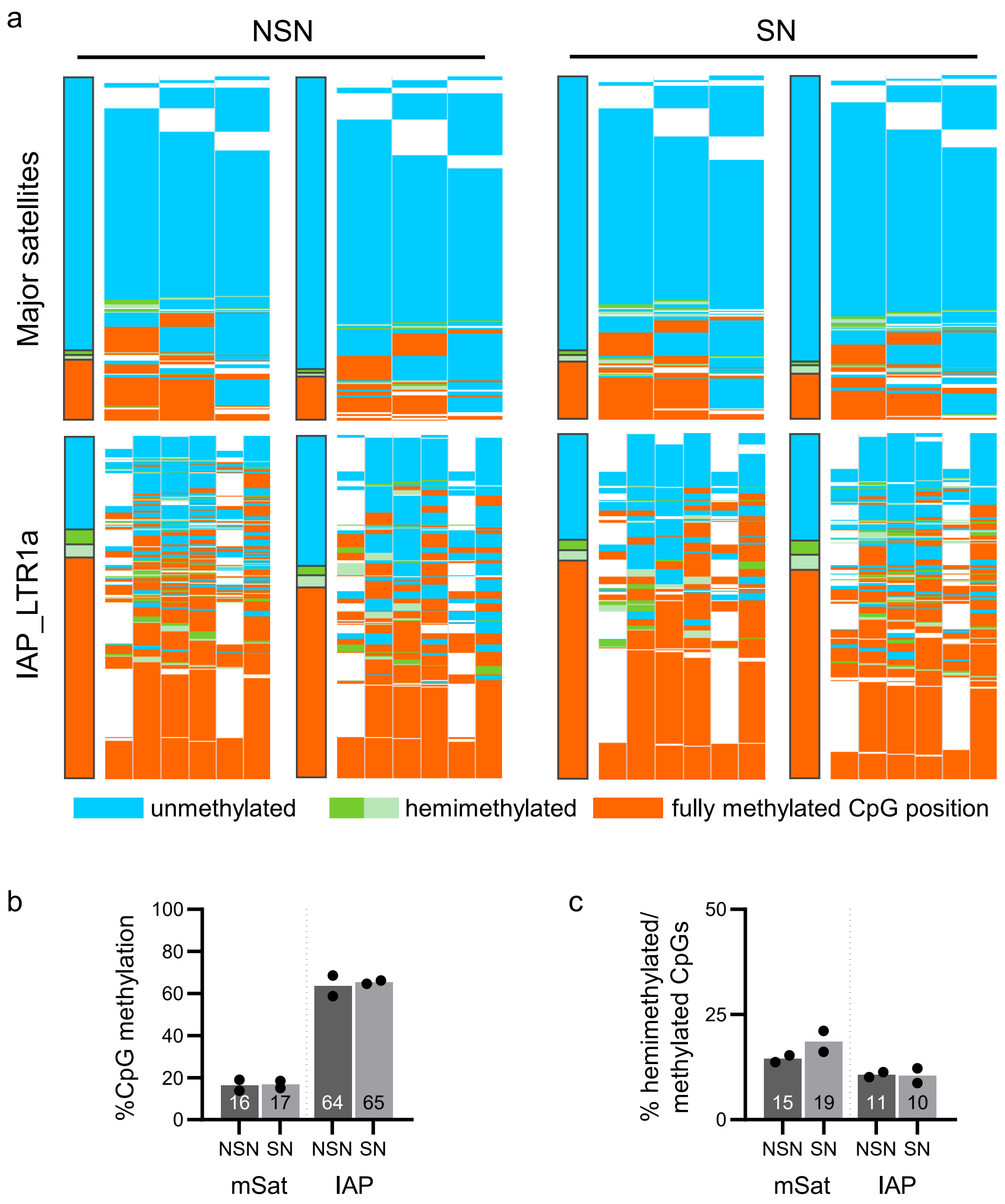

Supplementary Figure 4: DNA methylation of repeat elements in NSN- and SN-GVOs

(a) DNA methylation pattern map from hairpin bisulfite amplicon sequencing analysis. Each column represents neighbored CpG positions and each row one sequenced read. The bar on the left is the sum of unmethylated, hemi-methylated, or fully methylated CpG positions.

(b) Average methylation levels from panel a (n=2). **(c)** Average hemi-methylation levels from panel a (n=2) normalized to methylated sites.

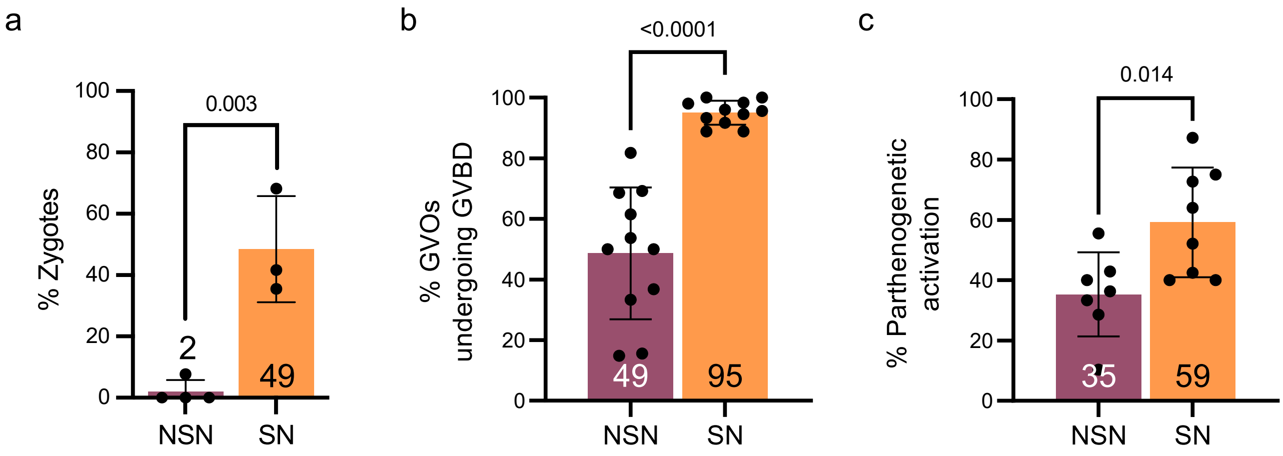

Supplementary Figure 5: *In vitro* fertilization, maturation, and parthenogenetic activation rates of NSN- and SN-GVOs

(a) Germinal vesicle breakdown (GVBD) oocytes derived from NSN- and SN-GVOs developing to zygotes (%) after *in vitro* maturation and *in vitro* fertilization. Each dot represents one independent experiment (n=4 for NSN and n=3 for SN). Statistical significance was calculated using Student's t-test; data are represented as mean ± SD.

(b+c) Progression rate from data in Figure 4a, subdivided by: **(b)** mean rate of GVOs undergoing GVBD and **(c)** mean rate of successfully activated parthenotes from MII-oocytes derived from NSN- or SN-GVOs. Each dot represents one independent experiment (b: n=11 for NSN and n=11 for SN; c: n=6 for NSN and n=8 for SN). Statistical significance was calculated using Student's t-test; data are represented as mean ± SD.

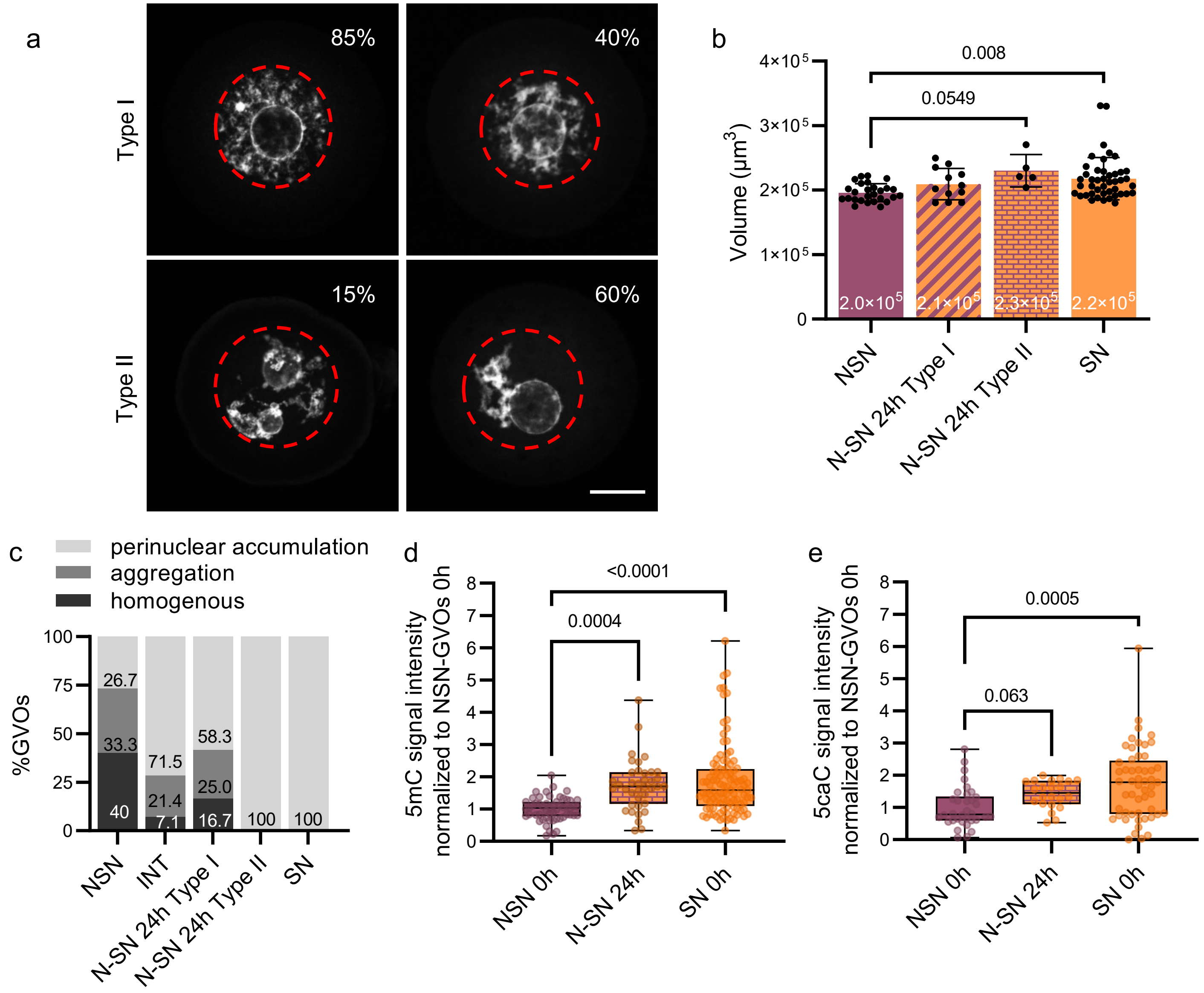

Supplementary Figure 6: Characterization of *ex vivo* transitioned SN-GVOs (N-SN-GVOs)

(a) Chromatin configuration of N-SN-GVOs shown by propidium iodide staining. Two types of N-SN-GVOs could be observed: Type I, with diffuse Hoechst staining in the nucleus, and type II, which resembles the natural SN-GVO chromatin conformation. After 48 h of culturing under maturation inhibiting conditions more type II N-SN-GVOs can be observed than after 24 h. The dashed lines indicate the nucleus. Scale bar = 20 µm.

(b) Quantification of GVO-volumes in the different types of GVOs. Each dot represents a single oocyte (NSN- and SN-GVO data from Figure 2; n=27 for NSN, n=12 for N-SN Type I, n=5 for N-SN Type II and n=44 for SN; data are derived from three independent experiments). Statistical significance was calculated using one-way ANOVA with Tukey's multiple comparison test; data are represented as mean ± SD.

(c) Mitochondrial localization pattern of N-SN-GVOs at 24 h.

(d+e) 5mC **(d)** and 5caC **(e)** antibody signal of N-SN-GVOs at 24 h. Signal intensity was normalized to NSN-GVOs in each experiment. Each dot represents a single oocyte (d: n=55 for NSN, n=38 for N-SN, n=104 for SN and data are derived from five independent experiment; e: n=34 for NSN, n=22 for N-SN, n=53 for SN and data are derived from nine independent experiments). The box plots are showing the interquartile (box), median (horizontal line), minimum and maximum values (error bars). Statistical significance was calculated using one-way ANOVA with Tukey's multiple comparison test.

a

	No. of oocytes examined	No. of GVOs undergoing GVBD	No. of GVBD oocytes developing to	
			1-cell stage	2-cell stage
**NSN-GVO 0h** n=4	97	25 (25.8%)[a]	1 (4%)[a]	0 (0%)
**N-SN 24h** n=4	47	13 (27.7%)[a]	3 (23.1%)[b]	0 (0%)
**N-SN type I 48 h** n=3	24	4 (16.7%)[a]	2 (50%)[c]	1 (25%)[a]
**N-SN type II 48 h** n=3	38	21 (55.3%)[b]	9 (42.9%)[c]	7 (33.3%)[a]
**SN-GVO 0h** n=5	126	111 (88.1%)[c]	89 (80.2%)[d]	69 (62.2%)[b]
**SN-GVO 48h** n=2	79	73 (92.4%)[c]	32 (43.8%)[c]	19 (26.0%)[a]

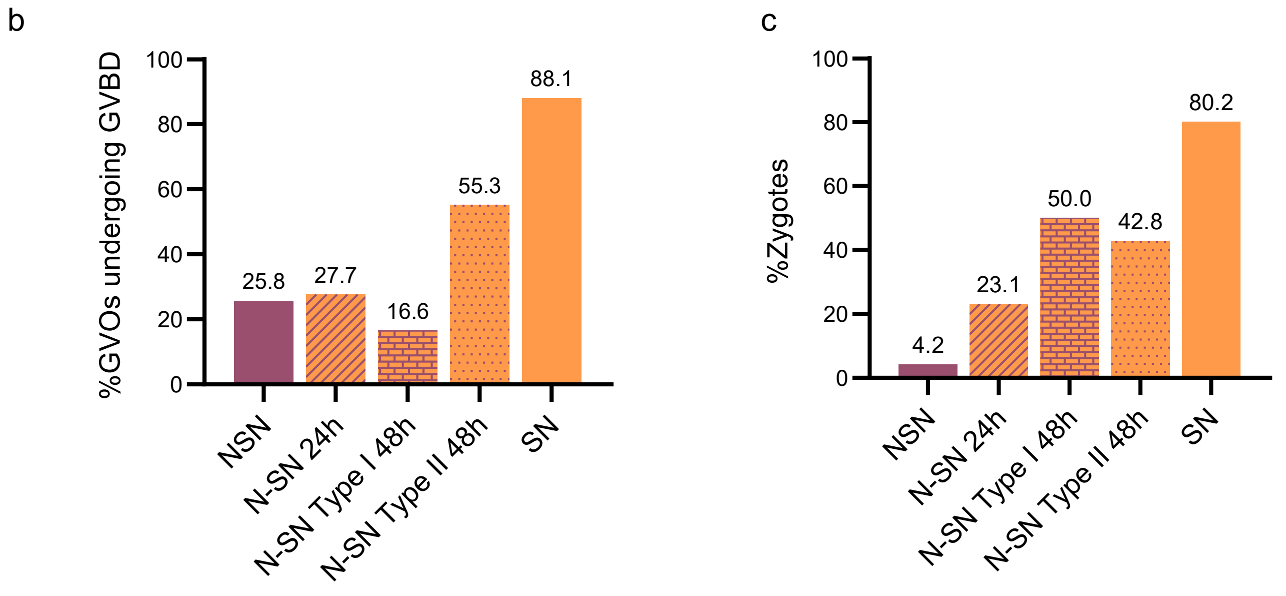

Supplementary Figure 7: Developmental competence of *ex vivo* SN-GVOs (N-SN-GVOs)

(a) Germinal vesicle breakdown (GVBD) and developmental competence of NSN-, N-SN-, and SN-GVOs. Statistical significance was calculated using χ^2 analysis. The superscripts indicate significant differences ($p<0.05$). The number of the independent experiments, from which the data are derived, is indicated in the first column.

(b+c) Visualization of percentages of **(b)** GVOs undergoing GVBD or **(c)** GVBD oocytes developing to zygotes after *in vitro* maturation and *in vitro* fertilization from **(a)**. Bars represent the percentage of total analyzed GVOs (see **(a)**).

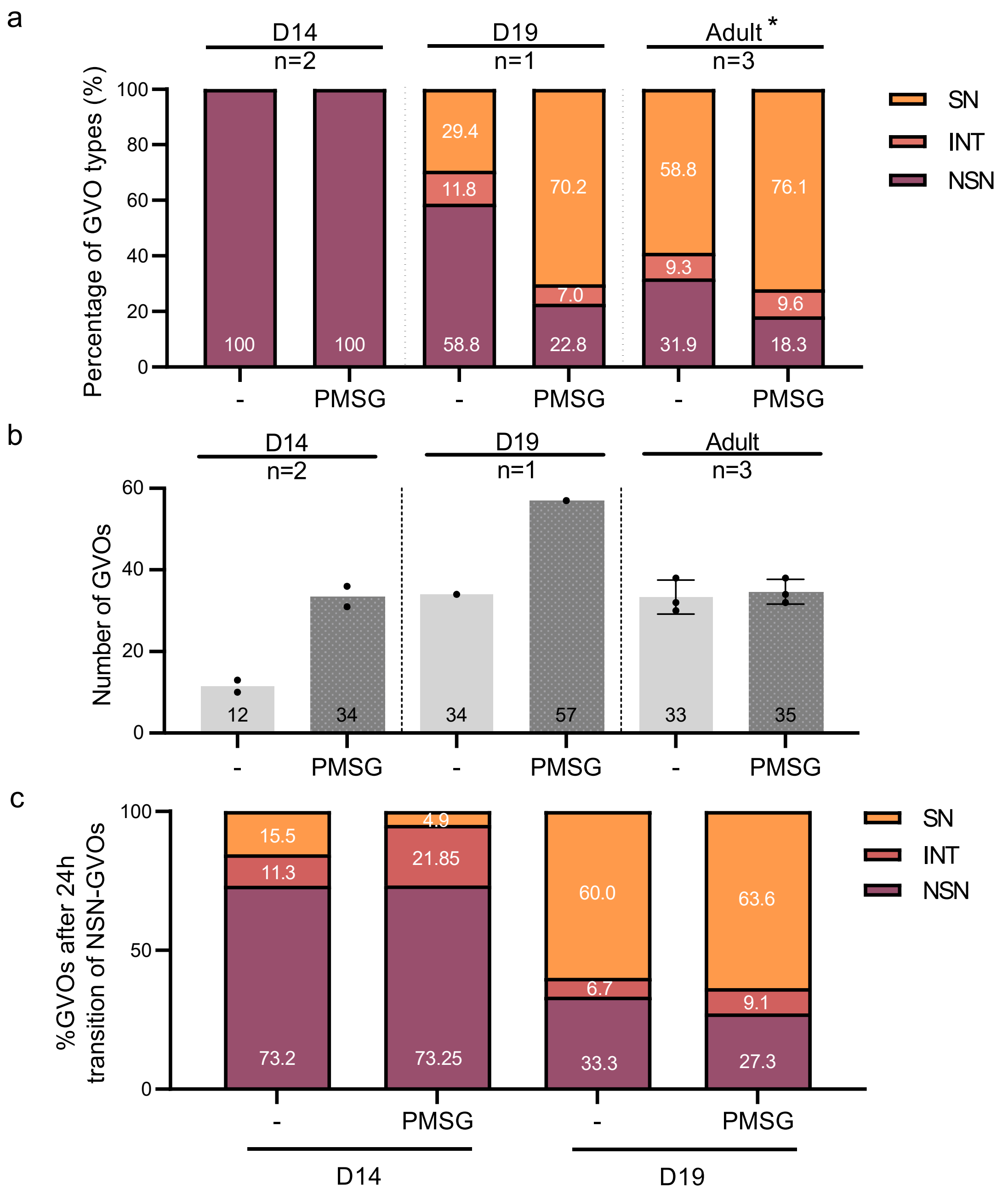

Supplementary Figure 8: Influence of PMSG on the occurrence of the different types of GVOs

(a) Percentage of the different GVO-types observed when freshly isolated from ovaries with (PMSG) or without (-) hormonal stimulation from young (D14 or D19) or adult mice (>8 weeks). Statistical significance for the NSN/SN ratio for the adult oocytes was calculated using χ^2 analysis (* = p=0.02).

(b) Number of GVOs per mouse with (PMSG) or without (-) hormonal stimulation from young (D14 or D19) or adult mice (>8 weeks). Each dot represents GVOs derived from one mouse. No significant statistical difference was observed in the NSN/SN ratio in adult mice (student's t-test; data are represented as mean ± SD).

(c) Percentage of different GVO types after 24 h culture of NSN-GVOs derived with (PMSG) or without (-) hormonal stimulation from D14 or D19 mice under *in vitro* maturation inhibiting conditions.

Supplementary Table 1: Distribution of the three types of GVOs in isolated mouse GVOs

**Distribution of the three types of chromatin configurations in mouse GVOs**			
	NSN	INT	SN
Percentage ± SD (%)	29.9±10.1	9.1±3.6	61.0±10.9
Total number of oocytes analyzed	572	167	1131
