# Supplementary material for: A transition phase in late mouse oogenesis impacts DNA methylation of the early embryo
Source: Commun Biol. 2022 Oct 2;5:1047. doi: 10.1038/s42003-022-04008-1 (PMC9527251; doi:10.1038/s42003-022-04008-1)
Supplement: Supplementary file 3 — Description of Additional Supplementary Files [file 42003_2022_4008_MOESM3_ESM.pdf]

Description of Additional Supplementary Files

File name: Supplementary Movie 1

Description: Time-lapse movie of germinal vesicle breakdown in NSN-GVO during in vitro maturation injected with H2B-YFP mRNA. Scale bar = 20 µm

File name: Supplementary Movie 2

Description: Time-lapse movie of germinal vesicle breakdown in SN-GVO during in vitro maturation injected with H2B-YFP mRNA. Scale bar = 20 µm.

File name: Supplementary Data 1

Description: Information on the sequencing reads from Supplementary Figure 4.

File name: Supplementary Data 2

Description: Source data behind the graphs in the paper.
